# Delays in Initiating Ambulance Transport Following Emergency Patient Referral to Mulago National Referral Hospital

**DOI:** 10.7759/cureus.115262

**Published:** 2026-08-26

**Authors:** Paul Omagor, Mary Ellen Lyon, Jessica Pelletier

**Affiliations:** 1 Anaesthesia, Critical Care and Emergency Medicine, College of Health Sciences, Makerere University, Kampala, UGA; 2 Emergency Medicine, University of Missouri-Columbia, Columbia, USA

**Keywords:** ambulance transport, emergency medical services (ems), inter-hospital transfers, low- and middle-income country (lmic), prehospital emergency medicine, resource-limited setting

## Abstract

Introduction

Timely ambulance transport is critical for emergency care, yet delays in initiating transport for transfers are common in low- and middle-income countries. In Uganda, given the limited ambulance fleet, we conducted a secondary analysis of data from a cross-sectional study that was conducted to assess compliance with national ambulance operational standards across key emergency medical services domains. The objective of this secondary analysis was to determine the factors associated with delays of more than 60 minutes in initiating ambulance transport from the decision to refer to Mulago National Referral Hospital.

Methods

This was a non-pre-specified secondary analysis of data from a cross-sectional study assessing ambulance operational performance in Uganda between May and August 2024. Variables in the primary dataset were reviewed for relevance to the specific outcome of delayed initiation of ambulance transport and were included in the analysis. The primary outcome was time to transport initiation following referral decision, categorized by a 60-minute threshold (≤60 vs >60 minutes), as per Uganda’s national EMS strategic plan for ambulance operations. Multivariable binary logistic regression analysis identified factors associated with delayed initiation.

Results

Among 118 emergency interfacility referrals, 71 (60.2%) were initiated after 60 minutes. Delayed initiation was observed more frequently when ambulances were occupied at the time of interfacility referral (AOR 5.5; p <0.008), fuel was inadequate (AOR 3.1; p 0.008), staffing was hospital-based rather than by dedicated emergency medical technicians (EMTs) (AOR 2.5; p 0.015), and when the availability of essential medical products was below 75% (AOR 2.4; p 0.032).

Conclusions

Most emergency interfacility referrals experienced delays in initiating ambulance transport. Delays were commonly observed in settings where ambulances were occupied, fuel was inadequate, staffing relied on hospital-based personnel, or medical products were limited. These findings identify operational factors associated with delayed ambulance transport initiation, highlight potential benchmarks for improving timeliness, and warrant further evaluation to refine time benchmarks for interfacility emergency transport.

## Introduction

Ambulance services are a critical component of health systems in providing rapid management and transportation of patients to appropriate medical facilities [1], directly improving time-sensitive patient outcomes [2,3]. Delays in ambulance response are associated with increased mortality among patients requiring urgent interfacility transfers [4,5]. The global approach to addressing ambulance response delays emphasizes coordinated dispatch systems, dedicated referral ambulances, and standardized ambulance readiness protocols [6,7]. Despite this, the development of emergency medical services (EMS) structures in low- and middle-income countries (LMICs) reveals persistent gaps in responsiveness, particularly where dispatch systems, standardized readiness protocols, equitable distribution, and access remain limited [8,9]. 

Although the importance of timely ambulance transport in emergency patient referral is well recognized in Sub-Saharan Africa (SSA), the expansion of ambulance fleet coverage in the region has not consistently been accompanied by proportional improvements in response intervals, and delays in emergency referral remain a persistent concern [10]. This observation suggests that challenges in emergency response may be attributable not only to fleet size but also to the organization, functionality, and integration of EMS systems within existing health structures [11,12]. Moreover, delays in ambulance emergency referral remain poorly described and insufficiently quantified in many SSA settings [12,13]. Hence, the limited empirical evidence characterizing these pre-transport intervals constrains health systems’ ability to identify modifiable bottlenecks and implement targeted readiness interventions. 

Uganda, whose health care delivery system is organized in a hierarchical structure from primary health care facilities to higher-level referral hospitals, faces ambulance referral delays that hinder emergency patient care [14,15]. The country's ambulance service is governed under the national EMS policy [16], with varied ownership (public, private-for‑profit, and private-not-for-profit providers) and serving both out-of-hospital pickups and interfacility transfers. Ambulance operations are coordinated regionally by the regional EMS coordinators (REC), and while privately owned ambulances operate independently, they are expected to adhere to national operational guidelines. Largely, operations rely on general health budgets, donor support, and institutional resources, with service coverage constrained, especially in rural areas. Call and dispatch systems are not yet fully operational or widely recognized by the public [14], and response time is variable, often affected by operational factors [16]. Of note, ambulances in Uganda are reserved for the transportation of patients requiring emergency care. 

The national EMS strategic plan sets a primary performance indicator: increasing the proportion of emergency patients receiving an ambulance response within one hour to at least 50% by 2025 [17]. Within this tiered referral system, we examined delays in initiating ambulance transport in Uganda, focusing on the impact of coordination mechanisms, resources, and readiness on emergency care interfacility referrals.

## Materials and methods

Study design and setting

The primary study was a cross-sectional assessment of ambulance operational performance for arrivals to Mulago National Referral Hospital between May and August 2024. We conducted a secondary analysis of these data to assess factors associated with delays in initiating transport following a decision to refer. This analysis focused on a 60-minute threshold for transport initiation following the referral decision, based on Uganda’s national EMS strategic plan for ambulance response times for both primary response and interfacility transfers [17]. The study was approved by the School of Medicine Research and Ethics Committee of Makerere University (Mak-SOMREC-2023-840), and only de-identified data were utilized. 

Sample size

The sample size (n) for this analysis was 118. This was the total number of ambulance emergency interfacility transfers conducted and available in the dataset. 

Inclusion and exclusion criteria

The study population included ambulances transferring emergency patients to the Accident and Emergency Department (A&E) of Mulago National Referral Hospital. We included all records for interfacility ambulance arrivals during the study period. We excluded 11 records representing out-of-hospital pickups to ensure the cohort focused strictly on interfacility transfers. No records were excluded due to incomplete data.

Data collection and study variables

Data were derived from a primary dataset collected using a structured questionnaire developed from Uganda's EMS strategic plan and national ambulance norms and standards (see Appendix for the full questionnaire). Referral time was obtained from the patient referral form, and departure time was obtained by interviewing the ambulance driver upon arrival.

The primary outcome was referral delay, defined as the interval between the documented referral time and the reported departure time, categorized as ≤60 minutes or >60 minutes. Independent variables available in the primary study dataset were first reviewed for relevance to the specific outcome of delayed initiation of ambulance transport. Variables operationally relevant to the referral and transport process were included in the multivariable analysis. These included: referral distance, time of referral, mode of payment, ambulance affiliation, coordination, staffing, and availability of fuel and medical products. Fuel was classified as inadequate when the operator reported insufficient fuel as the reason for the delay, and an ambulance was classified as preoccupied when the operator reported it was engaged in another response at the time of referral.

We utilized an investigator-derived Operational Readiness Score (ORS) to assess readiness. The ORS was calculated by summing four binary components (scored 1 if present, 0 if absent): dedicated EMT staff, adequate fuel, ambulance not preoccupied, and availability of ≥75% essential medical products.

Data analysis

Data were reviewed for completeness, and duplicate entries were removed before analysis. Statistical analyses were performed using Stata version 16/MP (StataCorp, College Station, TX, USA). Categorical variables were summarized as frequencies and percentages and reported as n (%). Associations between categorical variables and delayed ambulance response (>60 minutes) were assessed using Pearson's χ² test; Fisher's exact test was used when expected cell counts were <5. All variables included in the bivariate analysis were considered for inclusion in the multivariable binary logistic regression model. Results are reported as odds ratios (ORs) with corresponding 95% confidence intervals (CIs). Wald χ² statistics were used to test regression coefficients. Statistical significance was defined as a two-sided p-value <0.05.

## Results

**Figure 1:**
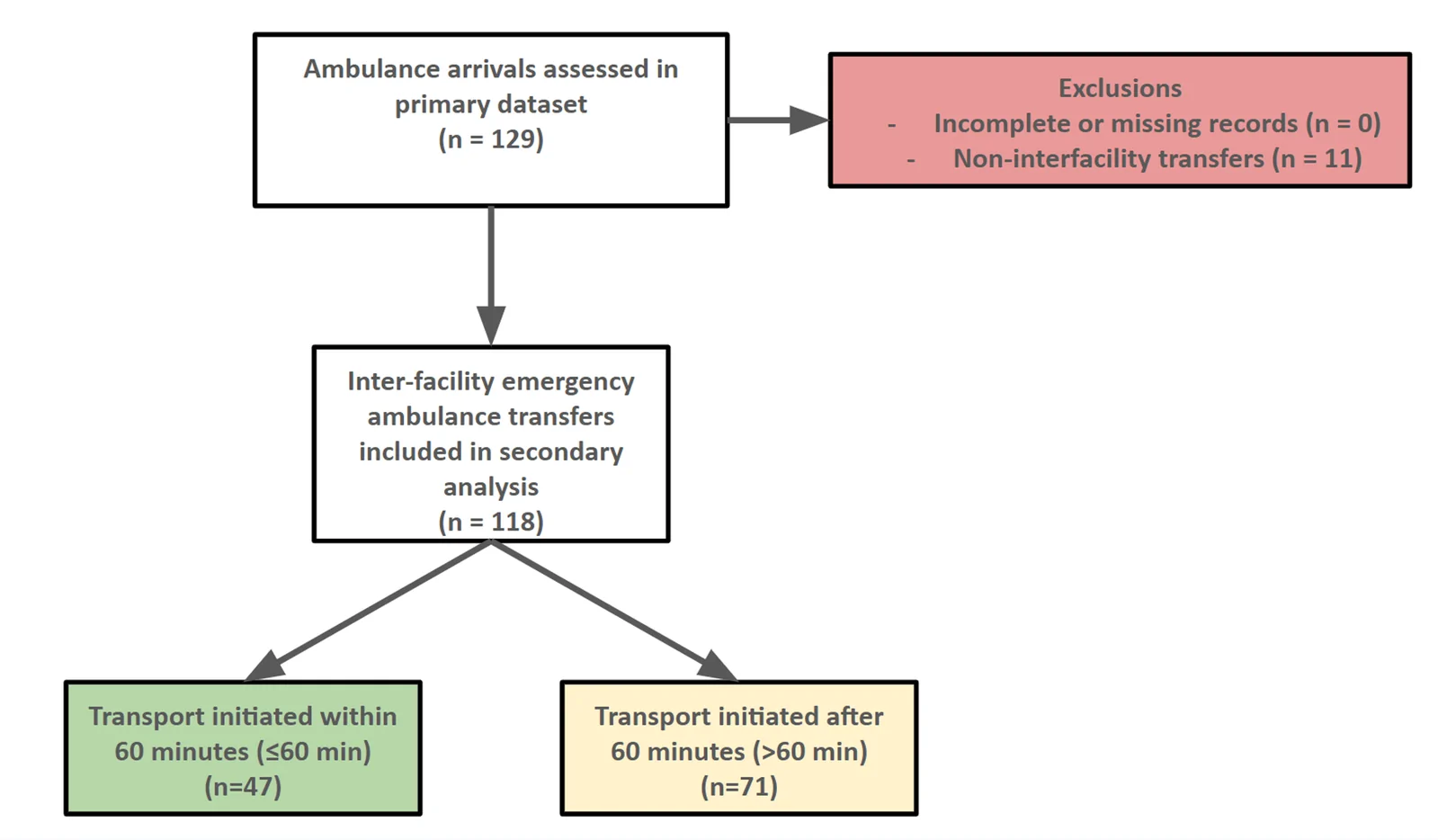
Study profile of ambulance arrivals and selection of inter‑facility emergency transfers for secondary analysis. Records representing out‑of‑hospital pickups were excluded prior to analysis.

There were 129 ambulance arrivals in the primary dataset. No records were excluded due to incomplete data. This left 118 interfacility transfers for inclusion in this secondary analysis (Figure 1).

Overall, 71 of 118 referrals (60.2%) experienced transport initiation delays exceeding 60 minutes. Delays were more frequent among referrals over distances >50 km (73.2% vs. 53.2%; χ² = 4.43, p = 0.035), ambulances staffed by hospital-based personnel rather than dedicated EMTs (69.4% vs. 45.7%; χ² = 6.63, p = 0.010), ambulances with inadequate fuel at the time of referral (69.3% vs. 33.3%; χ² = 12.09, p < 0.001), and ambulances that were preoccupied at the time of referral (74.0% vs. 34.1%; χ² = 17.75, p < 0.001). Medical products available in less than 75% of the recommended inventory were also associated with delayed transport initiation (Fisher's exact test, p = 0.025). No statistically significant associations were observed for day of referral, time of referral, type of payment, ambulance affiliation, or coordination by the REC (p > 0.05) (Table 1).

**Table 1:** Operational factors in initiating ambulance transport >60 minutes. EMT: Emergency medical technician; OOP: out-of-pocket payment; REC: regional emergency coordination center; PNFP: private not-for-profit; PFP: private for-profit. Statistical significance for categorical variables was determined using Pearson's Chi-square (χ²) test. For medical products, Fisher's Exact Test was used because one cell count was <5.

Variable	Category	n (%)	Initiated ≤60 min n (%)	Initiated >60 min n (%)	χ²	P-value
Overall	–	118 (100.0)	47 (39.8)	71 (60.2)	–	–
Interfacility referral distance	≤50 km	77 (65.3)	36 (46.8)	41 (53.2)	4.43	0.035
	>50 km	41 (34.7)	11 (26.8)	30 (73.2)		
Day of interfacility referral	Weekday	83 (70.3)	37 (44.6)	46 (55.4)	2.63	0.105
	Weekend	35 (29.7)	10 (28.6)	25 (71.4)		
Time of interfacility referral	Day	59 (50.0)	28 (47.5)	31 (52.5)	2.86	0.091
	Night	59 (50.0)	19 (32.2)	40 (67.8)		
Type of payment	Non-OOP	60 (50.8)	26 (43.3)	34 (56.7)	0.62	0.429
	OOP	58 (49.2)	21 (36.2)	37 (63.8)		
Ambulance affiliation	Public	54 (45.8)	19 (35.2)	35 (64.8)	0.91	0.636
	PNFP	27 (22.9)	12 (44.4)	15 (55.6)		
	PFP	37 (31.4)	16 (43.2)	21 (56.8)		
Coordination	REC	26 (22.0)	14 (53.8)	12 (46.2)	2.73	0.098
	Non-REC	92 (78.0)	33 (35.9)	59 (64.1)		
Staffing	Dedicated EMT	46 (39.0)	25 (54.3)	21 (45.7)	6.63	0.010
	Hospital-based personnel	72 (61.0)	22 (30.6)	50 (69.4)		
Fuel/gas at interfacility referral time	Adequate	30 (25.4)	20 (66.7)	10 (33.3)	12.09	<0.001
	Not adequate	88 (74.6)	27 (30.7)	61 (69.3)		
Ambulance availability	Preoccupied	77 (65.3)	20 (26.0)	57 (74.0)	17.75	<0.001
	Not preoccupied	41 (34.7)	27 (65.9)	14 (34.1)		
Medical products	≥75%	11 (9.3)	8 (72.7)	3 (27.3)	5.48†	0.025†
	<75%	107 (90.7)	39 (36.4)	68 (63.6)		

Ambulances staffed with hospital-based personnel other than a dedicated EMT were associated with a higher likelihood of ambulance transport delays exceeding 60 minutes (adjusted odds ratio, AOR 2.5, 95% CI 2.7 (1.2-6.1), p=0.015). Similarly, delays were more likely when fuel was inadequate at the time of interfacility referral (AOR 3.1, 95% CI 3.4 (1.4-8.0), p=0.008), when ambulances were preoccupied (AOR 5.5, 95% CI 5.8 (2.3-8.0), p<0.001), and when standard medical product inventory was below 75% (AOR 2.4, 95% CI 2.3 (1.7-7.3), p=0.032). Other factors, including interfacility referral distance, day and time of interfacility referral, type of payment, ambulance affiliation, and coordination type, were not statistically significant (Table 2).

**Table 2:** Factors associated with transport initiation >60 minutes after the decision to refer. COR: Crude odds ratio; AOR: adjusted odds ratio; CI: confidence interval; REC: regional emergency coordination center; OOP: out-of-pocket; PNFP: private not-for-profit; PFP: private for-profit. Odds ratios were estimated using binary logistic regression. Wald χ² statistics were used to test regression coefficients.

Category	n (%)	COR (95% CI)	AOR (95% CI)	Wald χ²	P-value
Interfacility referral distance >50 km	41 (34.7%)	2.3 (0.9-5.6)	1.6 (0.7-3.8)	1.27	0.26
Staffed by hospital-based staff	72 (61.0%)	2.7 (1.2-6.1)	2.5 (1.2-5.2)	5.92	0.015
Referred over the weekend	35 (29.7%)	1.8 (0.7-4.3)	1.5 (0.7-3.3)	1.07	0.30
Fuel inadequate at time of interfacility referral	88 (74.6%)	3.4 (1.4-8.0)	3.1 (1.3-7.2)	6.98	0.008
Referred at night hour	59 (50.0%)	1.9 (0.8-4.2)	1.6 (0.7-3.4)	1.32	0.25
Ambulance preoccupied	77 (65.3%)	5.8 (2.3-8.0)	5.5 (2.1-7.6)	12.01	<0.001
Service paid OOP	58 (49.2%)	1.3 (0.6-2.7)	1.2 (0.6-2.4)	0.32	0.57
Ambulance <75% medical products	107 (90.7%)	2.3 (1.7-7.3)	2.4 (1.1-5.2)	4.60	0.032
Ambulance affiliated to PNFP	27 (22.9%)	0.8 (0.3-2.4)	0.9 (0.3-2.3)	0.06	0.80
Ambulance affiliated to PFP	37 (31.4%)	0.9 (0.4-2.3)	1.0 (0.4-2.3)	0.00	0.97
Under non-REC coordination	92 (78.0%)	2.1 (0.8-5.4)	1.5 (0.6-3.7)	0.84	0.36

Timely transport initiation varied across readiness categories, with 23.1% of low-readiness (ORS = 0-1) ambulances initiating transport within 60 minutes compared with 52.6% of moderately ready (ORS = 2), 72.7% of highly ready (ORS = 3), and 66.7% of fully ready (ORS = 4) ambulances. Thus, compared with low readiness, the proportion of timely transport initiation was substantially higher at readiness scores of 4 and 3 (Table 3).

**Table 3:** Operational readiness score with ≤ 60-minute transport initiation. *The ORS was an investigator-derived composite measure developed from variables that demonstrated statistically significant association and calculated by summing four binary components: Dedicated EMT staff (Yes = 1, No = 0), Adequate fuel at time of interfacility referral (Yes = 1, No = 0), Ambulance not preoccupied at time of interfacility referral (Yes = 1, No = 0), Availability of ≥75% essential medical products (Yes = 1, No = 0), Score ranges from 0 (least ready) to 4 (fully ready).

Operational Readiness Score*	n (%)	Initiated ≤60 min n (%)	Initiated >60 min n (%)
0-1 (Low readiness)	52 (44.1)	12 (23.1)	40 (76.9)
2 (Moderate readiness)	38 (32.2)	20 (52.6)	18 (47.4)
3 (High readiness)	22 (18.6)	16 (72.7)	6 (27.3)
4 (Fully ready)	6 (5.1)	4 (66.7)	2 (33.3)

## Discussion

Timely ambulance response is essential for patients with time-sensitive interfacility referrals, with delays significantly affecting clinical outcomes and overall health system efficiency [12,13,18]. The first observation in this study is that a significant proportion (60.2%) of ambulances respond more than 60 minutes after the decision to refer. This finding highlights a critical gap in EMS responsiveness, a challenge commonly reported across LMICs [19-21]. Ambulances undertaking interfacility referrals over distances greater than 50 km were more than fourfold more likely to initiate transport late than those traveling ≤50 km. This may reflect the additional operational planning required for longer-distance transfers, including route assessment, confirmation of receiving facility readiness, fuel adequacy, and crew preparation before departure. The finding suggests that referral distance influences not only travel time but also the time required to mobilize an ambulance, highlighting the need for regional referral networks, strategically distributed ambulance stations, and effective dispatch systems to reduce delays for patients requiring transfer over longer distances. 

Ambulances staffed by hospital-based personnel were 2.5 times more likely to experience delays than those staffed by dedicated EMTs, underscoring the importance of prehospital role specialization for rapid responses. The magnitude of this association suggests that reliance on hospital-based personnel may introduce competing clinical responsibilities that delay ambulance mobilization, whereas dedicated EMT models provide greater operational availability at the point of referral. In agreement, studies suggest hospital-based staff are often embedded within facility workflows and clinical duties, which can delay disengagement at the time of interfacility referral and slow ambulance mobilization [22,23]. This finding aligns with evidence from systematic reviews of urban and suburban EMS systems in Asian countries [24] and in SSA [13,25], where reliance on dual-role clinicians has been associated with longer activation and departure times than services employing dedicated prehospital teams.

Another observation relates to ambulance availability at the time of interfacility referral. Ambulances already occupied were more likely to initiate transport after 60 minutes, highlighting the impact of competing demands on limited fleet capacity. This was the strongest operational association identified in the analysis, with preoccupied ambulances demonstrating approximately five-fold higher odds of delayed transport initiation. This finding emphasizes that ambulance availability is a fundamental component of EMS readiness. In systems with limited ambulance fleets, a referral decision alone may not translate into timely transport if available vehicles are committed elsewhere. Similar challenges have been reported in African metropolitan EMS systems, where high call volumes and inadequate fleet size result in delayed responses even when interfacility referral decisions are made promptly [26]. By contrast, many European EMS systems manage interfacility referrals through transport services that are operationally distinct from emergency response ambulances, thereby reducing competition for vehicles at the point of interfacility referral. This separation allows interfacility referral transport to be planned and coordinated without compromising emergency response readiness and limits delays attributable to vehicle unavailability [23,27]. The contrast underscores that the delays observed in this study are primarily structural, driven by limited ambulance capacity and the absence of dedicated interfacility referral transport, rather than by failures in coordination alone.

Fuel availability also played a critical role in transport initiation. Ambulances lacking adequate fuel at the time of interfacility referral were significantly more likely to be delayed, reflecting persistent logistical vulnerabilities within emergency transport systems in low-resource settings. The association between inadequate fuel availability and delayed transport initiation was substantial, with fuel-related limitations associated with more than three-fold higher odds of delay. This finding demonstrates that routine logistical systems, including fuel planning and supply mechanisms, can directly influence emergency transport performance. Studies from Nigeria and other SSA contexts have consistently identified fuel shortages and decentralized procurement processes as contributors to delayed ambulance activation [28,29]. By comparison, in the United States, public EMS systems operate under centralized logistics and protected emergency budgets, ensuring that fuel constraints rarely interfere with response intervals [30].

Furthermore, the limited availability of medical products contributed to delays. Ambulances carrying fewer than 75% of the required supplies, as per the norms and standards for ambulances in Uganda [31], had over twice the odds of delayed initiation, suggesting that operational preparedness extends beyond staffing and vehicles to include supply chain management. The association between inadequate medical product availability and delayed transport initiation suggests that equipment readiness may serve as a marker of broader ambulance operational preparedness. Strengthening ambulance supply systems may therefore improve both mobilization efficiency and the ability to provide appropriate care during transport. This observation aligns with other LMIC studies demonstrating that insufficient medical inventory can prolong prehospital intervals and compromise patient care [32,33].

Collectively, the ORS demonstrated a positive relationship between cumulative operational preparedness and transport timeliness. Ambulances with higher readiness scores had progressively higher proportions of transport initiations within 60 minutes, indicating that the combined availability of dedicated staffing, fuel, vehicles, and essential medical products contributes to improved EMS responsiveness. The progressive increase in timely transport initiation across low- to high-readiness ambulances suggests that improvements in multiple operational domains may have an additive effect on ambulance responsiveness. This finding reinforces the importance of strengthening EMS readiness as a system rather than addressing individual operational components in isolation. In agreement, studies from both African and high-income settings have demonstrated that composite measures of ambulance readiness and system preparedness are stronger predictors of timely response [33-35]. Together, these findings indicate that delayed ambulance transport in Uganda is strongly associated with modifiable operational constraints. The observed associations range from a 2.4-fold increase in delay with inadequate medical products to a 5.5-fold increase when ambulances were preoccupied, highlighting that improvements in EMS performance will require coordinated strengthening of workforce, fleet availability, logistics, and supply systems. 

One major issue in Uganda that can make adequate staffing of EMS vehicles challenging is the lack of a clear distinction between vehicles that are solely capable of transporting patients and those that can provide medical care. Although the Ministry of Health defines EMS as capable of providing emergency health services [36], in reality, only 30.8% of Ugandan ambulances have the appropriate equipment, medications, and trained staff to provide emergency care [14]. Greater clarity in labeling is needed to distinguish vehicles solely capable of transport from those that can provide true EMS.

In 2021, the Ugandan Ministry of Health released a National EMS Policy with an overall goal of ensuring universal access to and affordability of EMS in the country. Key priority areas noted in this document include leadership and governance, health workforce, and access to essential medicines. The leadership and governance priority area highlights the importance of national and regional coordination across the public and private sectors [36]. Stronger coordination - for example, through regional dispatch centers - could ensure that ambulances with higher ORS at a given time are dispatched for transport. The health workforce priority area indicates the need for dedicated emergency medicine and EMS personnel to meet national accreditation standards [36]. Ensuring that dedicated prehospital personnel with standardized training staff ambulances, and that hospital-based personnel do not accompany patients during ambulance transport, would be particularly important given that Uganda’s hospitals are critically understaffed [36]. Access to essential medicines for ambulance services is challenging without financial support [36]. Ideally, funding from government or non-governmental organizations should be allocated to ensure that EMS has the appropriate medical supplies to care for patients in the prehospital setting. The Ugandan Ministry of Health aims to bolster EMS leadership and governance across all levels of health service delivery by 2030. Successful restructuring of the aforementioned key priority areas may help address the critical gaps in EMS preparedness identified in this study and, ideally, alleviate the burden of delayed transports.

Limitations

This single-center, secondary cross-sectional analysis may not fully represent ambulance interfacility referral delays across Uganda. The four-month duration offers a snapshot that may overlook seasonal variations. Furthermore, the small sample size limits statistical power and increases the risk of overfitting. Dataset limitations, including the absence of variables such as clinical severity, facility availability, and organizational demands, mean that residual confounding cannot be excluded despite multivariable analysis. Additionally, recall bias is a potential concern, as transport times, even when cross-checked with caregivers, may have been recorded with delays. Finally, the research team-developed ORS requires external validation, and these findings may not generalize beyond similar resource-limited EMS systems.

## Conclusions

Most emergency interfacility referrals experienced transport initiation delays exceeding 60 minutes before ambulance transport began. Delayed initiation was independently associated with ambulance unavailability, inadequate fuel availability, absence of dedicated EMT staffing, and limited medical supplies. These findings highlight operational gaps in ambulance service readiness that warrant targeted quality improvement initiatives and further evaluation to strengthen emergency referral systems in Uganda.

## Appendices

**Table 4:** Operations of land ambulances assessment form Credit: Dr. Paul Omagor and Dr. Mary Ellen Lyon. A+B: Airway and breathing; AEA: Ambulance Emergency Assistant; ACLS: Advanced Cardiovascular Life Support; ATLS: Advanced Trauma Life Support; APLS: Advanced Pediatric Life Support; BTLS: Basic Trauma Life Support; DHIS2: District Health Information Software 2; EMS: Emergency Medical Services; ECA: Emergency Care Assistant; MOH: Ministry of Health; NGO: Nongovernmental organization; NPA: Nasopharyngeal airway; OPA: Oropharyngeal airway; PHTLS: Prehospital Trauma Life Support; PNFP: Private Not-For-Profit; REC: Regional EMS Coordinator; UGX: Ugandan shillings.

Region		
Pillar 1: EMS Service Delivery		
i. What is the class of ambulance	☐ B ☐ C ☐ Not classified	
ii. Time of response	☐ D ☐ N	
iii. Where was the patient picked from?	☐ Private hospital ☐ Government hospital ☐ Out of hospital ☐ Other - specify: ______	
iv. Where was this ambulance stationed at dispatch	☐ Hospital ☐ Dispatch center ☐ Field station ☐ Active response	
v. Average response time (ART) (minutes)	☐ <60 ☐ 60-120 ☐ >120	
Factors contributing to this state of response		
What influenced the choice to use this ambulance for this response?	☐ Only available ambulance ☐ Closest ambulance to patient ☐ What a patient could pay for ☐ Required as determined ☐ Other - specify: ______	
If ART >60 minutes, what are the reasons for delayed response time?	☐ Payment surety ☐ Bad weather ☐ Bad terrain ☐ Traffic ☐ Distant pickup ☐ Poor mapping ☐ Ambulance was preoccupied ☐ Lack of blue lights and siren ☐ Other - specify: ______	
Interviewer comments for this section		
Pillar 2: EMS Health Workforce		
i. What is the EMS crew configuration for this response?		
Class B	Class C	
☐ Driver ☐ ECA ☐ EMT ☐ Other - specify: ______ (Note minimum recommended crew: Driver, ECA, EMT)	☐ Regular driver as AEA ☐ ECA ☐ EMT ☐ RNO or MO ☐ Other - specify: ______ (Note minimum recommended crew: AEA, ECA, EMT, RNO or MO)	
ii. Is there a lead supervisor for this response?	☐ Regional EMS Coordinator ☐ Professional with ATLS/ACLS/APLS, and BTLS or PHTLS or equivalent ☐ Dispatch center ☐ Hospital admin ☐ Fleet manager ☐ Other - specify: ______	
Factors contributing to this state of response		
If no EMT for B, or EMT/RNO/MO for C onboard, why?	☐ Cost of certification ☐ Lack of trainers ☐ Flexible organization policy ☐ Lack of added benefits ☐ Other - specify: ______	
Interviewer comments for this section		
Pillar 3: EMS Information System		
i. Which number was called to access this ambulance?	☐ Private ☐ REC private ☐ Company dispatcher (pay) ☐ Company dispatcher (toll free) ☐ MOH dispatcher (pay) ☐ MOH dispatcher (toll free)	
ii. Who did you contact in this hospital before arriving with the patient?	☐ Hospital phone ☐ Receiving nurse ☐ Receiving doctor ☐ Hospital administration ☐ No contact was made ☐ Other - specify: ______	
Factors contributing to this state of response		
Does this ambulance have an allocated access number?	☐ Private ☐ REC private ☐ Company dispatcher (pay) ☐ Company dispatcher (toll free) ☐ MOH dispatcher (pay) ☐ MOH dispatcher (toll free) ☐ None	
Is the access number that was used branded on the ambulance?	☐ Yes ☐ No ☐ Different one from the one branded	
What are the reasons for not calling the hospital (ii)?	☐ Unknown hospital numbers ☐ Lack of prepaid calls ☐ Phone breakdown	
Interviewer comments for this section		
Pillar 4: EMS Medical Products		
Baseline	Class B	
Patient handling		
☐ Loading stretcher ☐ Carrying sheets	☐ Non-vacuum mattress	
Diagnostic equipment		
☐ Sphygmomanometer ☐ Pulse oximeter ☐ Stethoscope ☐ Thermometer ☐ Glucometer ☐ Pen light	☐ Monitor ☐ AED	
A+B		
☐ Suction device ☐ OPA + NPA ☐ Magill forceps ☐ Nebulization device ☐ Stationary oxygen ☐ Portable oxygen	As baseline	
Infusion material		
☐ Infusion solutions ☐ Glucose ☐ Cannulas ☐ Infusion set ☐ Infusion mounting ☐ Gauze ☐ Bandages	As baseline	
Immobilization equipment		
☐ Traction device ☐ Fracture splints ☐ Cervical collar ☐ Spinal board	As baseline	
PPE and safety		
☐ Reflective jackets ☐ Gloves ☐ Safety helmet ☐ Disinfection material ☐ Warning triangle ☐ Fire extinguisher	As baseline	
Factors contributing to this state of response		
Reasons for not meeting the required products	☐ Cost of product ☐ Not aware of the requirement ☐ Lack of personnel to use ☐ Transit safety of the products ☐ Other - specify: ______	
Interviewer comments for this section		
Pillar 5: Financing		
i. What is the mode of payment for this response?	☐ Insurance prepaid ☐ No direct pay ☐ <100,000 UGX ☐ 100,000-500,000 UGX ☐ 500,000-1,000,000 UGX ☐ >1,000,000 UGX	
Factors contributing to this state of response		
Interviewer comments for this section		
Pillar 6: Governance		
i. Ownership	☐ Public ☐ Private ☐ PNFP ☐ NGO ☐ Other - specify: ______	
ii. Ambulance approved by MOH EMS office?	☐ Yes ☐ No	
iii. Which platform do you feed in your response outcomes?	☐ DHIS2 ☐ Company platform ☐ Other - specify: ______	
Factors contributing to this state of response		
If not (i) approved, why?	☐ Approval fee ☐ Lack of information ☐ No licensure given ☐ Other - specify: ______	
Interviewer comments for this section		
